# Relative fat mass assessment estimates changes in adiposity among female older adults with obesity after a 12-month exercise and diet intervention

**DOI:** 10.1080/07853890.2022.2067352

**Published:** 2022-04-26

**Authors:** Katelyn E. Senkus, Kristi M. Crowe-White, Julie L. Locher, Jamy D. Ard

**Affiliations:** a Department of Human Nutrition, The University of Alabama, Tuscaloosa, AL, USA; b Division of Gerontology, Geriatrics and Palliative Care, Department of Medicine, University of Alabama at Birmingham, Birmingham, AL, USA; c Department of Epidemiology and Prevention, Wake Forest University School of Medicine, Winston-Salem, NC, USA

**Keywords:** Relative fat mass, total body fat, older adults, dual-energy X-ray absorptiometry, cardiometabolic disease

## Abstract

**Background/objectives/introduction:**

It is imperative to accurately estimate whole body fat percentage (%fat) to understand the deleterious nature of excess adiposity on cardiometabolic disease risk. Cost and accessibility often preclude the use of advanced imaging methods like dual-energy X-ray absorptiometry (DXA) and magnetic resonance imaging (MRI). Relative fat mass (RFM) is an emerging estimator of whole body %fat based on waist circumference, height, and biological sex. The purpose of this ancillary study was to examine the relationship between RFM and gold-standard measures of adiposity among community-dwelling older adults with obesity and to evaluate if changes in RFM reflect changes in %fat following a 12-month lifestyle intervention (clinicaltrials.gov #NCT00955903).

**Patients/materials and methods:**

Participants (*N* = 163, 37.4% male, 70.3 ± 4.7 years) were randomized to the exercise only group, exercise + nutrient-dense weight maintenance group, or exercise + nutrient-dense energy restriction of 500 kcal/d group. Total and regional adiposity assessed by DXA and MRI, as well as anthropometrics, were evaluated at baseline and 12 months.

**Results:**

RFM was significantly positively correlated with DXA whole body %fat and DXA trunk %fat at baseline. Equivalence testing revealed that RFM was considered equivalent to DXA whole body %fat for females only. Additionally, from baseline to 12 months, a significant reduction in RFM was observed among female participants in the exercise + energy restriction group only. Changes in RFM were significantly correlated with changes in DXA whole body %fat, DXA trunk fat, and total abdominal fat tissue determined by MRI.

**Conclusion:**

Results support the use of RFM as an estimate of whole body %fat where advanced imaging techniques are not feasible. Furthermore, results suggest that this index is sensitive to changes in fat mass over 12 months in female older adults with obesity.

**KEY MESSAGES**
- Relative fat mass (RFM), an emerging estimator of whole body %fat based on waist circumference, height, and biological sex, was intentionally developed to be a simple estimate of adiposity that overcomes limitations of measures like body mass index.
- In the current study, results from correlations and agreement analyses support the use of RFM to estimate whole-body fat percentage in a community-dwelling older adult population with obesity when advanced methods, namely dual-energy X-ray absorptiometry, are not feasible.
- Significant reductions in RFM were also observed over a 12-month period that was significantly correlated with changes in whole body fat percentage; thus, supporting the sensitivity of RFM to lifestyle changes.

## Introduction

It is well-established that obesity, a condition characterized by the accrual of excess adipose tissue (AT), increases cardiometabolic disease risk and development [1]. AT is an active endocrine organ and with excess adiposity, there is a dysregulation of adipokine secretion followed by deleterious metabolic changes, including inflammation, redox imbalance, and insulin resistance, among others [2,3]. Acknowledging that 42% of Americans are currently living with obesity, it is critical to determine optimal interventions that will beneficially influence cardiometabolic disease risk [4]. To achieve such goals, simple, yet reliable methods to accurately estimate whole body fat percentage (%fat) are needed.

Imaging modalities, such as dual-energy X-ray absorptiometry (DXA), computed tomography (CT), and magnetic resonance imaging (MRI), are gold standard methods for the assessment of body composition [5]. DXA provides a non-invasive assessment of fat mass and fat-free mass, allowing for the determination of %fat. This can also be quantified using CT and MRI while concurrently discerning between visceral and subcutaneous adipose tissue depots [2]. However, cost and accessibility often preclude the use of these advanced assessment methods. In attempts to overcome such limitations, various indexes have been developed for the evaluation of body composition, namely body mass index (BMI). Although routinely employed in clinical and research settings, BMI is also limited in its ability to differentiate between lean mass and fat mass and its association with fat mass varies by biological sex, age, ethnicity, and disease [5].

Relative fat mass (RFM) is an emerging index used to estimate whole body %fat based on waist circumference, height, and biological sex [6]. The development and validation of this index were completed using a nationally representative sample of United States adults and the authors concluded that RFM more accurately estimated whole body %fat than BMI. Similar findings have been reported for this index among children, young adults, and individuals with Down Syndrome; however, it has yet to be evaluated among older adults with obesity [7–9]. Additionally, its sensitivity to intervention-driven change has yet to be investigated. As such, the purpose of this study was to examine the relationship between RFM and gold-standard measures of adiposity among community-dwelling older adults with obesity and to evaluate if changes in RFM reflect changes in %fat following a 12-month lifestyle intervention.

## Materials and methods

### Participants

This study was ancillary to a randomized controlled trial conducted by Ard et al. between 2009 and 2014 that investigated the effects of a 12-month exercise and diet intervention among older adults with obesity and at risk for cardiometabolic disease (Calorie Restriction in Overweight SeniorS: Response of Older Adults to a Dieting Study, CROSSROADS Study, ClinicalTrials.gov #NCT00955903) [10]. Community-dwelling men and women ages 65 years or older were recruited from the Birmingham, Alabama, USA area using various advertisements and word-of-mouth recruitment methods. Participants were required to be at a high risk of cardiometabolic disease as determined by a BMI indicative of Class 1 or 2 obesity (30–40 kg/m^2^) and taking at least one medication to control lipids, blood pressure, or blood glucose. Eligibility was assessed at multiple time points before enrolment (one telephone and three in-person screening visits) and exclusion criteria included medical, physical, or psychiatric limitations that would prevent intervention adoption and/or confound lifestyle-related body weight changes. All participants provided written informed consent for the parent study and this ancillary analysis. Protocols were approved by the Institutional Review Board at The University of Alabama at Birmingham and The University of Alabama.

### Study design

Briefly, participants were assigned to one of three intervention groups for 12 months: (1) Exercise only, (2) Exercise + nutrient-dense weight maintenance (exercise plus weight maintenance), or (3) Exercise + nutrient-dense caloric restriction of 500 kcal/d (exercise plus weight loss). A block randomization scheme was employed using a computer-based algorithm stratified by biological sex, age (65–74, 75+ years), and race, and group assignment was concealed in opaque envelopes until randomization occurred. All participants adhered to a standard aerobic and resistance training exercise program. Participants in the exercise only group met with a registered dietitian nutritionist (RDN) at baseline and received written instructions for following a healthy diet. Participants in the exercise plus weight maintenance and exercise plus weight loss groups received counselling by an RDN throughout the intervention period to improve dietary quality with recommendations grounded in the time-calorie displacement theory. Additionally, exercise plus weight maintenance and exercise plus weight loss groups were given daily calorie goals based on estimates of total energy expenditure obtained from the measured resting metabolic rate at baseline. Data were collected at baseline and 12 months by blinded study personnel. Further study design details have been extensively described elsewhere [11–13].

### Anthropometrics

Standing height was measured without shoes to the nearest 0.1 cm using a manual stadiometer. Waist circumference was also measured to the nearest 0.1 cm at the narrowest point above the iliac crest and below the xyphoid process with a flexible tape measure. The following equation was used to determine the relative fat mass (RFM):

$$64-(20\times (\frac{\mathrm{Height}\;(m)}{\mathrm{Waist}\;\mathrm{Circumference}\;(m)}))\;+(12\times \mathrm{Biological}\;\mathrm{Sex})$$

where male and female are coded as 0 and 1, respectively [4].

### Assessment of body composition

Fat mass (total and trunk) was determined using dual-energy X-ray absorptiometry (DXA) with a Lunar DPX-L densitometer and Adult Software Version 1.33 (Lunar Corp, Madison, WI, USA). Abdominal adipose tissue (intra-abdominal, subcutaneous, and total abdominal volume) was assessed by magnetic resonance imaging using a 3-Tesla Phillips Achieve System (Philips, Andover, MA, USA).

### Statistical analysis

Baseline data were analyzed using Spearman’s correlation and linear regression analyses to evaluate the relationship between RFM and gold-standard measures of adiposity. Agreement between RFM and whole body %fat determined by DXA was assessed using Bland-Altman plots and 95% equivalence testing, herein referred to as equivalence testing [14,15]. For equivalence testing, the clinically significant error was defined as 10% and used to determine the equivalence region for the reference measure (DXA). A 90% confidence interval (CI) for the surrogate measure (RFM) was calculated and compared to the aforementioned equivalence region. To achieve statistical equivalence (*α* = 5%), the 90% CI for RFM must fall completely within the 10% equivalence region of mean DXA whole body %fat. Within-group and between-group changes in RFM at 12 months were evaluated using Wilcoxon signed-rank tests and Kruskal-Wallis tests with *post-hoc* analysis and Bonferroni correction for multiple comparisons, respectively. All analyses were performed using SPSS version 25 (SPSS Inc., Chicago, IL, USA). Variables are presented as mean ± standard deviation (*SD*) or median (25th percentile, 75th percentile) depending on normality assessment.

## Results

### Study participants

This ancillary analysis consisted of 163 participants (70.3 ± 4.7 years, 37.4% male, 23.9% African American). Participant characteristics are presented in Table 1. Briefly, participants had an average BMI of 33.7 kg/m^2^ (Class 1 obesity) and an average RFM of 41.2%. Female participants exhibited a significantly higher RFM compared to males [45.2% (43.6, 47.4%) and 33.9% (32.7, 36.1%), respectively (*p* < .001)]. Participants were randomized to the exercise only group (*n* = 54), the exercise plus weight maintenance group (*n* = 55), or the exercise plus weight loss group (*n* = 54).

**Table 1.** Descriptive characteristics of participants.

Variable	All participants	Males	Females
Age (years)	70.3 ± 4.7	70.5 ± 4.8	70.2 ± 4.7
Male sex, no. (%)	61 (37.4)	–	–
Race/ethnicity, no. (%)			
European American	124 (76.1)	55 (90.2)	69 (67.6)
African American	39 (23.9)	6 (9.8)	33 (32.4)
Weight (kg)	95.0 ± 14.1	105.8 ± 12.0	88.5 ± 11.0
Height (m)	1.7 ± 0.1	1.8 ± 0.1	1.6 ± 0.1
BMI (kg/m^2^)	33.7 ± 3.0	33.7 ± 3.2	33.6 ± 3.0
WC (cm)	111.2 ± 11.4	119.1 ± 8.3	106.4 ± 10.3
DXA whole body %fat (%)	45.9 ± 6.1	40.0 ± 4.3	49.5 ± 3.6
RFM (%)	41.2 ± 5.9	34.2 ± 2.1	45.4 ± 2.6

BMI: body mass index; DXA: dual-energy X-ray absorptiometry; RFM: relative fat mass; WC: waist circumference; %fat: percent fat.

Data are presented as mean ± *SD*.

### Characterizing the relationship between RFM and gold-standard measures of adiposity at baseline

**Figure 1.**
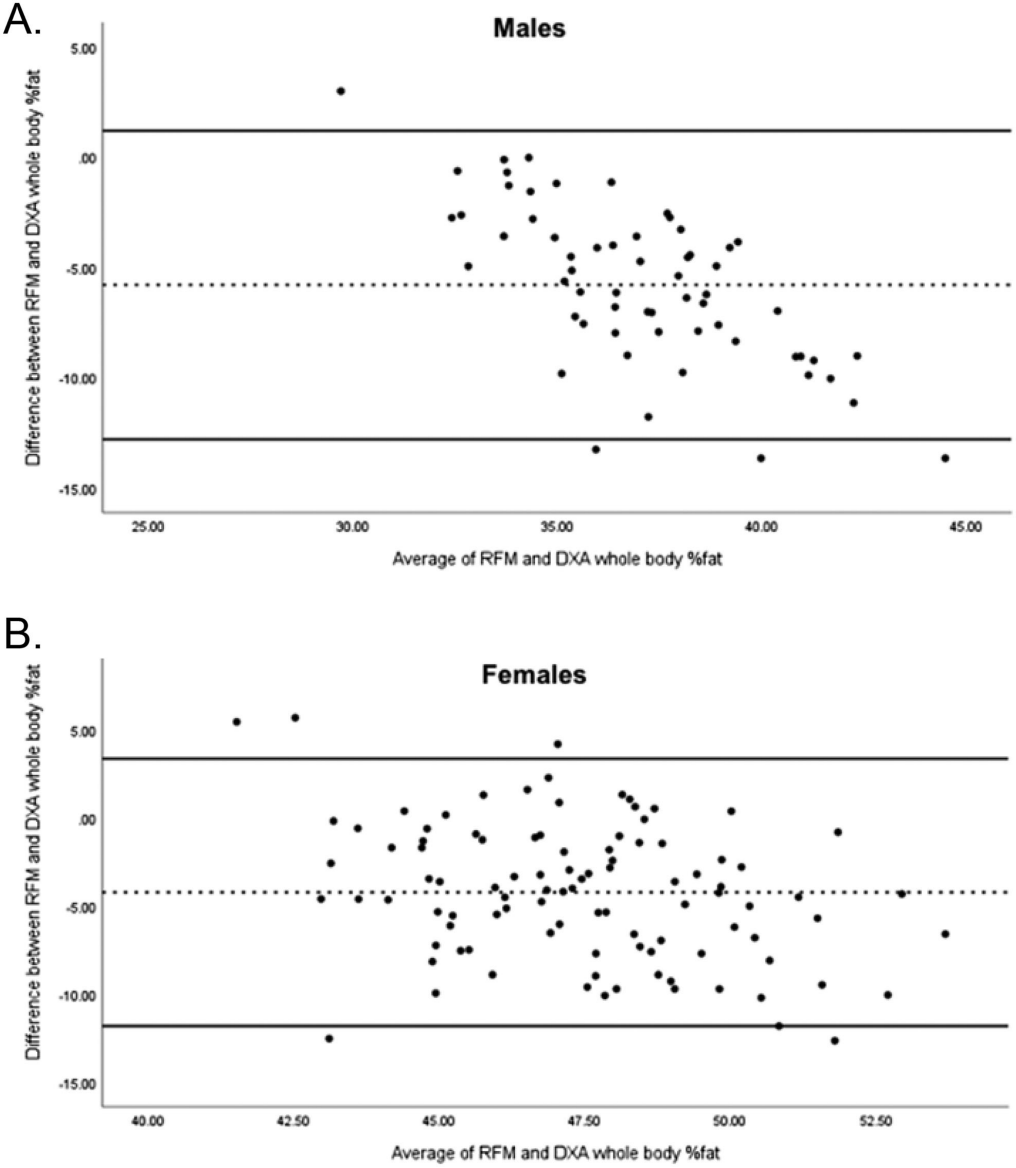
Bland-Altman plots assessing the agreement between relative fat mass (RFM) and whole body percent fat (%fat) determined by dual-energy X-ray absorptiometry (DXA) in (A) males and (B) females. The middle dashed line represents the mean bias and the upper and lower lines represent the limits of agreement (LOA) of ±1.96 standard deviations (*SD*). In males, the mean bias ± *SD* between RFM and DXA whole body %fat was −5.80 ± 3.6% and the LOA was −12.8, 1.2%. In females, the mean bias ± *SD* between RFM and DXA whole body %fat was −4.2 ± 3.9% and the LOA was −11.8, 3.4%.

Among all participants, RFM was significantly positively correlated with DXA whole body %fat and DXA trunk %fat (*r* = 0.751, *p* < .001; *r* = 0.661, *p* < .001, respectively). Additionally, RFM was a significant predictor of DXA whole body %fat (*p* < .001) and accounted for 63.5% of the model variance. Bland-Altman plots are presented in Figure 1. An inverse correlation was observed for males (Figure 1(A)), suggesting that RFM underestimates whole body %fat in individuals with higher total body fat. In contrast, the differences for females were more evenly distributed around the mean (Figure 1(B)).

For RFM and DXA whole body %fat to achieve equivalence, the 90% CI for RFM must fall completely within the 10% equivalence region of mean DXA whole body %fat (Equivalence region for all participants: 41.3–50.5%; Equivalence region for males: 36.0–44.0%; Equivalence region for females: 44.6–54.5%). Among all participants and among males only, RFM estimates were not completely inside the equivalence region (90% CI for all participants: 40.4–41.9%; 90% CI for males: 33.7–34.6%). However, among females, the RFM estimate was completely inside the equivalence region (90% CI for females: 44.9–45.8%); thus, the surrogate (RFM) and reference (DXA) methods were deemed significantly equivalent.

### Changes in RFM following a 12-month exercise and diet intervention

No significant differences in RFM were observed among groups at baseline. From baseline to 12 months, only female participants in the exercise plus weight loss group exhibited a significant reduction in RFM (*p* = .004) (Table 2). Similar within-group changes were observed for male participants in the exercise plus weight loss group, yet, did not achieve statistical significance (*p* = .055). The magnitude of RFM change did not differ among groups. Changes in RFM were weakly-to-moderately correlated with changes in adiposity measures, notably DXA whole body %fat (*r* = 0.279, *p* = .001), DXA trunk %fat (*r* = 0.276, *p* = .001), and total abdominal fat tissue determined by MRI (*r* = 0.404, *p* < .001).

**Table 2.** RFM by group at baseline and after 12 months of a lifestyle intervention with and without weight loss.

	Control		Weight maintenance		Weight loss	
	Baseline	Completion	Baseline	Completion	Baseline	Completion
Males						
RFM	34.3 (32.7, 36.3)	33.4 (32.9, 35.3)	34.4 (33.1, 35.9)	34.1 (32.6, 35.9)	33.4 (31.9, 36.1)	32.3 (31.2, 33.8)
*p*-Value		*p* = .093		*p* = .170		*p* = .055
Females						
RFM	46.1 (44.0, 47.9)	44.8 (42.4, 47.9)	44.9 (43.3, 46.8)	45.5 (42.9, 47.5)	45.2 (43.6, 47.4)	43.7 (42.5, 46.0)
*p*-Value		*p* = .064		*p* = .710		***p* = .004**

RFM: relative fat mass; completion = 12-months.

Data are presented as median (25th percentile, 75th percentile). All analyses conducted separately by biological sex.

Included *p*-values are related to comparison of RFM values at baseline and completion within the same group using Wilcoxon Signed Ranks test (i.e. baseline weight loss value *vs.* completion weight loss value). Statistical significance was defined as *p* < .05 and is represented by bold.

## Discussion

This study aimed to examine the relationship between RFM and gold-standard measures of adiposity among community-dwelling older adults with obesity and to evaluate if changes in RFM reflect changes in %fat following a 12-month lifestyle intervention. Strong correlations between RFM and DXA measures were observed among all participants at baseline, and assessment of agreement suggests that RFM is significantly equivalent to DXA whole body %fat among female participants only. Additionally, significant reductions were observed over a 12-month period that were significantly correlated with changes in DXA whole body %fat.

It is imperative to accurately estimate whole body %fat to understand the deleterious nature of excess adiposity on cardiometabolic disease risk. Various measures have been developed to assess body composition, including skin fold assessment, BMI, and equations like CUN-BAE and Gallagher [5,16]. However, these measures consistently fall short as their accuracy varies considerably by population, or their complexity limits clinical implementation [5,6]. RFM was intentionally developed to be a simple estimate of whole body %fat using anthropometric variables that can easily be collected in a clinical or research setting [6]. Using nationally representative data from the National Health and Nutrition Examination Survey (NHANES), this index was determined to better predict whole body %fat, measured by DXA, compared to BMI. The present study builds upon the validation study such that it investigates RFM among biracial, community-dwelling older adults with obesity and at high risk for cardiometabolic disease. Results demonstrated that in this population, RFM was significantly associated with DXA whole body %fat and trunk %fat. Similar correlations between RFM and DXA have been reported in generally healthy American and Brazilian adults [8,17].

The current study also employed equivalence testing to assess agreement between the measures of RFM and DXA whole body %fat. This statistical approach is recommended when comparing a new method to an established criterion as it is designed to detect equivalence rather than differences and overcomes limitations associated with other statistical methods like linear regression [15,18]. Among all participants, RFM was not considered equivalent to DXA whole body %fat. Subgroup analysis revealed that RFM was considered significantly equivalent to DXA whole body %fat among female participants only. In the original validation study by Woolcott et al., differences in the performance of RFM by biological sex were not reported; however, it is plausible that differences in the study samples influenced results [6]. For example, the average age of participants in the current analysis was 70 years old compared to ∼45 years old of those in the validation study. Nonetheless, results from the current study warrant further investigation into potential differences in the accuracy of RFM by biological sex. A recent study conducted by Fedewa et al. also used equivalence testing to evaluate the validity of RFM in a group of healthy, young adults [8]. Interestingly, RFM did not achieve equivalence for the total group or subgroup analysis by biological sex, despite strong correlations with whole body %fat as determined by four-compartment (4 C) modelling. These discrepancies underscore the importance of employing multiple methods to assess agreement rather than a single approach.

To date, much of the literature surrounding RFM is comprised of validation studies in different populations [7–9,19]. Such research is critical to understanding the generalizability of this index; however, it is also important to investigate the sensitivity of RFM to lifestyle interventions. In the current study, significant reductions in RFM were observed over a 12-month period in females of the exercise plus weight loss group. These changes were weakly-to-moderately correlated with changes in DXA whole body %fat. It should be noted that in the parent study, changes in DXA whole body % fat ranged from 0.3 to 1.6% across the three intervention groups [10]. Acknowledging that RFM did not have a perfect agreement with DXA measures, it is possible that changes in whole body %fat are not wholly reflected in changes in RFM. This should not detract from the use of RFM, as overall results support its ability to estimate whole body %fat among a female older adult population with obesity and at high risk for cardiometabolic disease. Given the novelty of this index, additional research is needed to evaluate its clinical usefulness among male older adults, as well as to evaluate the sensitivity of RFM to change in other populations.

DXA, CT, and MRI are preferred techniques for body composition analysis [2,5]. Both CT and MRI allow for a precise cross-sectional evaluation of compartmental body composition, thus, discerning between visceral and subcutaneous adipose tissue depots. DXA remains valuable as a three-compartment model that measures the fat mass, lean body mass, and bone mineral content for the total body and various body regions. It has been demonstrated to have a good correlation with CT and MRI fat measures with the added bonus of being a low-dose radiation technique. Nevertheless, all three imaging modalities are costly and require trained professionals to perform and analyze the output. Furthermore, accessibility remains a primary barrier, as reimbursement restrictions severely limit the use of DXA in primary care settings [20]. Results from the current study suggest that RFM can be used to estimate whole body %fat in settings where advanced imaging modalities are not feasible. This index, based on waist circumference, height, and biological sex only, may bolster the health care practitioner’s understanding of patient health as it relates to total adiposity; thus, influencing recommendations and care to promote cardiometabolic health. Given the relationship between excess adiposity, cardiometabolic disease, and mortality, it is important to define RFM thresholds that can easily identify high-risk individuals. Woolcott et al. recently proposed that an RFM above 30% for men or 40% for women reflects the need for immediate lifestyle intervention [21]. Continued investigation of these thresholds and their relationship with cardiometabolic disease risk is warranted.

Strengths of this study include robust outcome measures, namely the use of DXA and MRI to assess adiposity. To our knowledge, this is also the first study to evaluate RFM as a measure of adiposity in a biracial, community-dwelling, older adult population with obesity and at high risk for cardiometabolic disease, as well as to evaluate its sensitivity to lifestyle interventions. Although this study provides insightful results, it is not exempt from limitations. DXA is a three-compartment model routinely used to assess body composition in research; however, others have noted it to be inferior to the 4 C model to estimate body composition. Additionally, acknowledging that this was an ancillary analysis of a randomized controlled trial, the sample size and heterogeneity may obscure some relationships. Nevertheless, results from this exploratory study are important to guide future research in the validation and implementation of RFM among an older adult population.

## Conclusions

Overall, results support the use of RFM to estimate whole body %fat in a community-dwelling, female older adult population with obesity and at high risk for cardiometabolic disease. Additionally, results suggest that RFM is sensitive to change over a 12-month period of intervention with weight loss. Incorporation of RFM in clinical practice and cardiometabolic research may provide meaningful information not reflected in conventional body composition measures, such as BMI.

## Data Availability

The data that support the findings of this study are available from the corresponding author, KMCW, upon reasonable request.
